# The effect of animal grazing on vegetation and soil and element cycling in nature

**DOI:** 10.1007/s11356-017-0740-5

**Published:** 2017-11-21

**Authors:** Krzysztof Głowacz, Roman Niżnikowski

**Affiliations:** 1 Faculty of Animal Sciences, Department of Animal Environment Biology, Warsaw University of Life Sciences—SGGW, Ciszewskiego 8, 02-786 Warsaw, Poland; 2 Faculty of Animal Sciences, Department of Animal Breeding and Production, Warsaw University of Life Sciences——SGGW, Ciszewskiego 8, 02-786 Warsaw, Poland

**Keywords:** Sheep, Extensive grazing, Pasture sward botanical composition, Macro- and microelements, Bio-elements

## Abstract

Appropriate level of bio-components in blood plasma of animals is associated with their concentration in soil and in green fodder. Cycling of elements in nature and their adequate level in animal organisms result in proper functioning of an organism as a whole (Khan et al. 68:279–284, 2007). Therefore, it is important to analyse soil and green fodder for these components. If some deficits are found, it is important to supplement components responsible for e.g. proper growth and development of a young organism, proper reproduction and, consequently, good quality of obtained product, which may affect human health (Minzanova et al. 134:524–533, 2015). The aim of the presented study was to demonstrate the effect of extensive grazing by sheep on vegetation and soil and to analyse the cycling of some elements important for animal health. The effects of vegetation season on the concentration of potassium in soil and differences in the concentration of some elements in soil, green fodder and animal blood were demonstrated.

## Introduction

The usefulness of lands for grazing is affected by many factors. Most important is soil, its mineral components and their availability for plants. Availability of soil components is determined by: grain size structure, pH, water and air conditions, redox potential, organic matter content and appropriate fertilisation (Warda et al. 1996; Gustafson et al. 2003). In lands not used by agriculture, soil is fertilised with faeces of grazing animals (Groberek and Niżnikowski 2003, Groberek et al. 2004). In alternative systems of animal production, soil may become impoverished in mineral components necessary for plants (Groberek and Niżnikowski 2003). Nitrogen, magnesium and copper are the elements of high mobility, which may thus be easily washed out of soil (Šoch et al. 2011). Copper concentration is particularly important for small ruminants, because it is associated with wool production (Isamov et al. 2014). Phosphorus may easily combine with other elements (like Ca, Fe, Al) to produce hardly soluble salts less available for plants. Soil factors, botanical composition and vegetation period affect in turn mineral content of pasture vegetation; therefore, it is so important to provide appropriate soil fertilisation (Łozicki and Dymnicka 2003).

Appropriate level of elements in soil and green fodder determines normal functioning of animal organism and their status may be checked by analysing concentrations of selected biochemical indices in blood plasma (Mikhailowa et al. 2000; Łozicki and Dymnicka 2003). Deficit or excess of a given element may be the reason of many diseases. The amount of Cu, Zn, Se, Ca, P and Mg in animal blood depends on feeding, age and possible infections (Orr et al. 1990; Soder and Stout 2003, Kuba et al. 2015, Kwiecień et al. 2016).

The aim of the performed experiment was the analysis of flow of some macro- and microelements in the system soil—green fodder—blood plasma of animals. Demonstration of relationships between particular components of the flow system and concentration of elements in selected study material may improve animal health by supplementing possible deficits.

## Material and methods

### Study area, selection of animals

Studies were performed in northern part of Wielkopolskie Voivodship in areas where extensive grazing of sheep of the Wrzosówka Polska race was practiced for 15 or so years. The content of selected macro- and microelements was analysed in soil, green fodder and in blood of two groups of sheep: dams and lambs.

## Chemical analyses

### Soil analysis

Soil samples were taken with the Egner’s cane in the beginning and end of the study period from places used for collecting green fodder. Combined sample taken from each quarter was analysed for the concentration of: Ca, P, Mg, K, Cu, Na and Zn. Analyses were made in Analytical Centre SGGW in Warsaw. Sodium, potassium, magnesium calcium, zinc and phosphorus were determined with the ICP-AES method (Polish norm PB34 4th ed. of 11. April 2008) and copper—with the FAAS method (Polish norm PB10 5th ed. of 4 April 2008). Soil pH was determined in the same periods.

### Analysis of green fodder

Chemical analysis of green fodder was made in the laboratory of Department of Animal Nutrition and Biotechnology, SGGW in Warsaw. With the standard method (AAOC 1990), basic chemical composition of green fodder was determined. Analysed components included dry mass, total protein, crude fat, crude ash, crude fibre and its fractions: ADF (Acid Detergent Fibre) and NDF (Neutral Detergent Fibre) and ADL (Acid Detergent Lignin). Fibre fractions were determined with the Van Soest et al.’s (1991) method using Fibre Tec apparatus.

### Analysis of blood plasma in sheep

To estimate the track of elements from soil through green fodder to animal blood, samples of blood were taken from external jugular veins from the same animals in the beginning and end of the pasture season. Blood sample was then centrifuged to obtain plasma. Analysed blood was taken from dams and lambs. Samples of plasma were analysed for the same macro- and microelements as in soil and green fodder and in the same Analytical Centre. In total, 44 samples of blood plasma from animals were analysed.

### Statistical analyses of results

To calculate the concentration of mineral components in soil, green fodder and plasma, mean values of analysed material were calculated for each study period and year. In case of finding significant effect of studied material on analysed properties, the significance of differences was checked with Duncan test (Ruszczyc 1981).

Correlations were calculated between the concentration of particular elements in studied material and between botanical composition of sward and selected elements in green fodder from pasture. All statistical calculations were made with the SPSS 14.0 (2009) software.

## Results

### The concentration of mineral components in soil

The effect of selected factors and interaction on the concentration of mineral components in soil from study area is presented in Table 1.

**Table 1:** The effect of selected factors and interactions on chemical composition of soil (*N* = 48)

Component	Factors			Interactions			x̅	s.e.
	Sampling site	Year	Vegetation period	Sampling site * year	Sampling site * vegetation period	Year * vegetation period		
Ca (mg/kg)	NS	X	NS	NS	NS	NS	265.04	23.9
Cu (mg/kg)	NS	XX	NS	NS	NS	NS	3.42	0.39
K (g/kg)	NS	XX	NS	NS	NS	NS	1.40	0.09
Mg (mg/kg)	NS	XX	NS	X	NS	X	21.52	0.88
Na (mg/kg)	XX	XX	NS	XX	NS	NS	0.62	0.03
Zn (mg/kg)	NS	XX	NS	NS	NS	NS	27.61	1.49
P (mg/kg)	NS	NS	NS	NS	NS	NS	0.55	0.05

X—*p* ≤ 0.05; XX—*p* ≤ 0.01; *NS* not significant

Performed analyses showed highly significant effect of sampling site on sodium concentration in soil. The site had no effect on other elements. Study year did not differentiate phosphorus concentration in soil but had significant effect on soil calcium and highly significant effect on the concentration of the remaining elements. Vegetation period did not affect any of the analysed elements. There was a highly significant effect of the interaction of sampling site and study year on sodium concentration in soil. Significant effect on magnesium concentration in soil was found for the interaction sampling site*year and year *vegetation period. Both interactions did not affect the concentration of other elements.

### The effect of vegetation period on the concentration of selected mineral components in soil

The effect of vegetation period on the concentration of analysed elements in the beginning and at the end of vegetation period is presented in Table 2.

**Table 2:** The effect of vegetation period on the concentration of selected mineral components in soil (*N* = 48)

Component		Vegetation period	
		May	September/October
Ca (mg/kg)	x̅	279.75	245.42
	s.e.	31.59	36.48
Cu (mg/kg)	x̅	3.31	3.57
	s.e.	0.52	0.6
K (g/kg)	x̅	1.13	1.15
	s.e.	0.12	0.14
Mg mg/kg)	x̅	23.72	18.59
	s.e.	1.16	1.34
Na (mg/kg)	x̅	0.79	0.58
	s.e.	0.03	0.03
Zn (mg/kg)	x̅	32.20	21.47
	s.e.	1.98	2.28
P (mg/kg)	x̅	0.52	0.59
	s.e.	0.07	0.08

Concentrations of calcium, magnesium, sodium and zinc were higher in the beginning of the season while those of potassium and phosphorus were higher at the break of September and October. No significant differences were found in the concentration of all elements between the beginning and the end of the growing season.

### The effect of studied factors and their interactions on the chemical composition of pasture sward

The effect of studied factors and their interactions on the chemical composition of pasture sward is presented in Table 3.

**Table 3:** The effect of studied factors and their interactions on the chemical composition of pasture sward (*N* = 48)

Component	Factors			Interactions			x̅	s.e.
	Sampling site	Year	Vegetation period	Sampling site *year	Sampling site *vegetation period.	Year* vegetation period		
Ca (mg/kg)	XX	XX	XX	NS	X	NS	74.85	2.48
Cu (mg/kg)	NS	XX	NS	NS	NS	NS	4.82	0.19
K (g/kg)	NS	XX	X	NS	NS	XX	0.94	0.04
Mg (mg/kg)	NS	XX	NS	NS	NS	NS	210.05	14.26
Na (mg/kg)	NS	XX	NS	NS	X	NS	3.87	0.52
P (mg/kg)	X	XX	NS	NS	NS	NS	57.30	4.81
Zn (mg/kg)	NS	XX	NS	NS	NS	NS	3.31	0.14

There was a highly significant effect of year on the concentration of analysed elements in fodder. Significant effects of sampling site and vegetation period on Ca concentration, of sampling site on P concentration and of vegetation period on K concentration were noted. Interaction of sampling site*vegetation period significantly affected Ca and Na concentration in pasture sward. Highly significant was the effect of year*vegetation period on potassium concentration and significant was the effect of sampling site*vegetation period on sodium concentration.

### Concentrations of chemical components of pasture plants during the growing season

Nutrient contents in green fodder varied during the growing season (Table 4). The highest concentrations of K, Mg and Zn were noted at the break of June and July, those of Ca, P and Na at the break of September and October and of Cu in May. Concentrations of Ca, Cu, P and Zn were lowest in June and July, of K and Mg in May and of Zn at the end of the growing season.

**Table 4:** The effect of the growing season on chemical composition of pasture sward (*N* = 48)

Component		Growing season		
		May (A)	June/July (B)	September/October (C)
Cu (mg/kg)	x̅	4.89	4.72	4.85
	s.e.	0.33	0.33	0.33
K (g/kg)	x̅	0.81	1.11	0.89
	s.e.	0.07	0.07	0.07
	*	B.C	A	A
Mg (mg/kg)	x̅	204.20	221.26	204.67
	s.e.	24.70	24.70	24.70
Na (mg/kg)	x̅	4.51	2.44	4.67
	s.e.	0.90	0.90	0.90
P (mg/kg)	x̅	57.38	53.00	61.54
	s.e.	8.33	8.33	8.33
Zn (mg/kg)	x̅	3.20	3.56	3.19
	s.e.	0.25	0.25	0.25

*A, B, C—*p* ≤ 0.01

### Botanical composition and the content of some elements in green pasture fodder

Relationships between botanical composition of green fodder and the content of some elements are presented in Table 5. Highly significant negative correlation was found between the contribution of grasses to green fodder and percent of legumes, other dicotyledons and sedges and rushes. Similar relationship was noted between the share of aster family and other dicotyledons and between Ca concentration and percentage of legumes. No other correlations were found between the concentration of analysed elements and botanical composition of green fodder. Positive significant correlation was noted between percent of horsetails and other dicotyledons and between percent of sedges and rushes and that of legumes.

**Table 5:** Coefficients of significant correlations between various taxa of pasture vegetation and between the share of particular taxon and the concentration of selected elements (*N* = 64)

Components	Grasses (%) *Poaceae (%)*	Aster family (%) *Asteraceae (%)*	Legumes (%) *Fabaceae (%)*	Other dicotyledons dwuliścienne (%) *Dicotyledoneae (%)*	Horsetails *Equisetaceae (%)*	Sedges and rushes (%) *Cyperaceae et* Juncaceae (%)	Ca (mg/kg)
Grasses (%) *Poaceae* (%)			− 0.50 **	− 0.46 **		− 0.88 **	
Aster family (%) *Asteraceae* (%)				− 0.38 **			
Legumes (%) *Fabaceae* (%)	− 0.50 **					0.47 **	− 0.39 **
Other dicotyledons (%) *Dicotyledoneae* (%)	− 0.46 **	− 0.38 **			0.25 *		
Horsetails *Equisetaceae* (%)				0.25 *			
Sedges and rushes (%) *Cyperaceae et* Juncaceae (%)	− 0.88 **		0.47 **				

*p ≤ 0.05

**p ≤ 0.01

### Determination of the concentration of elements in soil, green fodder and blood plasma

Differences in the concentration of elements in soil, green fodder and blood plasma are presented in Table 6.

**Table 6:** Concentrations of elements in soil, greed fodder and blood plasma

Element		Analysed material			
		Soil (A) *N* = 32	Green fodder (B) *N* = 32	Blood plasma of dams (C) *N* = 16	Blood plasma of lambs (D) *N* = 28
Ca (mg/kg)	x̅	425.31	743.45	249.94	251.09
	s.e.	11.89	11.89	13.74	13.74
	*	B,C,D	A,C,D	A,B	A,B
Cu (mg/kg)	x̅	3.81	3.69	2.58	2.34
	s.e.	0.51	0.51	0.59	0.59
K (g/kg)	x̅	1.55	0.96	1.35	1.21
	s.e.	0.18	0.18	0.21	0.21
Mg (mg/kg)	x̅	58.02	100.23	15.09	15.54
	s.e.	29.87	29.87	3.45	3.45
Na (mg/kg)	x̅	3.37	4.28	3.67	3.48
	s.e.	0.3	0.3	0.35	0.35
P (mg/kg)	x̅	169.56	136.69	174.85	184.10
	s.e.	8.73	8.73	10.08	10.08
	*	B	A,C,D	B	B
Zn (mg/kg)	x̅	2.63	2.79	1.41	1.51
	s.e.	0.23	0.23	0.27	0.27
	*	c,d	C,D	a,B	a,B

*a, ..., d—*p* ≤ 0.05; A,…,D—*p* ≤ 0.01

Highly significant difference was observed in calcium concentration between soil and green fodder and concentration of this element in blood plasma of both dams and lambs. Phosphorus concentration in green fodder differed significantly from that in soil and blood plasma. Significant difference was found in zinc concentration between soil and blood plasma of dams and lambs and highly significant difference—between Zn in green fodder and in blood plasma.

## Discussion

Soil is an important factor that plays various functions. It is responsible for water flow regulation; as a natural buffer, it degrades xenobiotic compounds, and most importantly, for animal performance—it is a medium for plant growth and development (Franzluebbers 2002). Appropriate content of macro- and microelements in soil affects in part their content in green fodder (Xu et al. 2006, Xin et al. 2011).

Demand for nutritional components in animals depends on age, mode of nutrition and production, and the interaction of these factors. The same is true for macro- and microelements. Their concentration in bodily liquids of animals may evidence animal condition but also proper fodder value and indirectly—the soil, from which the fodder is harvested (McDowell 2003). Blood plasma may be the index reflecting the health status of an animal. As shown by Khan et al. (2007), concentrations of selected components of plasma depend on the mode of feeding, species composition of green fodder and even on the season of sampling. We found similar relationship when comparing composition of soil, green fodder and blood plasma of dams and lambs of sheep race Wrzosówka grazing on fallow lands. Significant differences were noted in the concentration of Ca, P and Zn in analysed material. Concentrations of all elements in blood plasma of dams and lambs were similar to the results obtained by McDowell (2003), Xin et al. (2011), but lower than concentrations reported by Khan (2003).

Both sheep and goats eat plants selectively depending in part on plant chemical composition (Hadjigeorgiou et al. 2003). Selectivity is an outcome of different demand of those two groups of animals for energy, protein, crude fibre and also for some macro- and microelements. As shown in our study, concentration of these components may vary in relation to the growing season. Obtained results differed from concentrations recommended by Petron et al. (2007) and Mayberry et al. (2010). Concentrations of Na, Ca and K were three times lower, and concentrations of P, Zn and Cu were two times lower in analysed green fodder than those recommended by both cited authors. Only the concentration of Mg might be considered appropriate.

## Conclusions

1. Negative correlation was found between percent of legumes and concentration of Ca in green fodder. With increasing percent share of legumes in sward, calcium concentration decreased.
2. Concentrations of selected elements did not differ between blood plasma of dams and lambs. Significant or highly significant differences were found in concentrations of Ca, P and Zn between soil, green fodder and blood plasma. The highest concentration of calcium and zinc was noted in green fodder and the lowest in blood plasma of animals. The reverse tendency was found for phosphorus.
3. Performed studies indicate the need of monitoring concentrations of particular elements in soil and green fodder. Results of monitoring should serve as a basis for balancing concentrations of these elements in blood of grazing animals.
